# Sequence diversity in three tomato species: SNPs, markers, and molecular evolution

**DOI:** 10.1186/1471-2229-9-85

**Published:** 2009-07-03

**Authors:** José M Jiménez-Gómez, Julin N Maloof

**Affiliations:** 1 Department of Plant Biology, College of Biological Sciences, University of California Davis, Davis, CA, 95616, USA

## Abstract

**Background:**

Tomato species are of significant agricultural and ecological interest, with cultivated tomato being among the most common vegetable crops grown. Wild tomato species are native to diverse habitats in South America and show great morphological and ecological diversity that has proven useful in breeding programs. However, relatively little is known about nucleotide diversity between tomato species. Until recently limited sequence information was available for tomato, preventing genome-wide evolutionary analyses. Now, an extensive collection of tomato expressed sequence tags (ESTs) is available at the SOL Genomics Network (SGN). This database holds sequences from several species, annotated with quality values, assembled into unigenes, and tested for homology against other genomes. Despite the importance of polymorphism detection for breeding and natural variation studies, such analyses in tomato have mostly been restricted to cultivated accessions. Importantly, previous polymorphisms surveys mostly ignored the linked meta-information, limiting functional and evolutionary analyses. The current data in SGN is thus an under-exploited resource. Here we describe a cross-species analysis taking full-advantage of available information.

**Results:**

We mined 20,000 interspecific polymorphisms between *Solanum lycopersicum *and *S. habrochaites *or *S. pennellii *and 28,800 intraspecific polymorphisms within *S. lycopersicum*. Using the available meta-information we classified genes into functional categories and obtained estimations of single nucleotide polymorphisms (SNP) quality, position in the gene, and effect on the encoded proteins, allowing us to perform evolutionary analyses. Finally, we developed a set of more than 10,000 between-species molecular markers optimized by sequence quality and predicted intron position. Experimental validation of 491 of these molecular markers resulted in confirmation of 413 polymorphisms.

**Conclusion:**

We present a new analysis of the extensive tomato EST sequences available that represents the most comprehensive survey of sequence diversity across *Solanum *species to date. These SNPs, plus thousands of molecular makers designed to detect the polymorphisms are available to the community via a website. Evolutionary analyses on these polymorphism uncovered sets of genes potentially important for the evolution and domestication of tomato; interestingly these sets were enriched for genes involved in response to the environment.

## Background

Tomato (*Solanum lycopersicum*) is the second most popular vegetable crop in the world [1]. In addition, tomato is being developed as a model organism and is more closely related to important crops like lettuce and coffee than other models such as Arabidopsis, poplar or rice [2]. Tomato also has an interesting natural history, which includes a single domesticated species and a number of wild relatives that have wide morphological variability and are adapted to very diverse environments [3,4]. The comparative study of tomato wild species can help us identify key genetic factors involved in domestication and will benefit breeding programs.

Despite the advent of high throughput genomic techniques and bioinformatics, comprehensive genome-wide information remains unavailable for most species, including tomato. Since the tomato genome sequence is currently incomplete and the microarray platforms for this species do not feature most of the loci predicted to exist [5-7], genetic studies have focused on the analysis of particular loci and segregating populations. These studies led to in-depth information on a few loci and uncovered the potential usefulness of the natural variation existing in related species [3,4]. A major goal for tomato geneticists is the acquisition of comprehensive genome-wide information that can be used in the improvement of resistance, quality, aspect, flavor and growth in cultivated varieties [8].

A large amount of genetic and molecular information is available for tomato, most of which has been deposited at the SGN http://sgn.cornell.edu. In this database there are more than 320,000 ESTs from several tomato species. This abundant sequence repository has been used to develop polymerase chain reaction-based (PCR-based) molecular markers to build on the original, time consuming and cost ineffective restriction fragment length polymorphisms (RFLPs) [9-12]

Recently, bioinformatic surveys of genome sequences have revealed the importance of SNPs in shaping evolution and also in serving as molecular markers [13]. In tomato, initial genomic work on SNP discovery involved de novo sequencing of EST libraries and comparison to the existing databases [14], or faster and less expensive computer-aided mining of the available sequences [15,16]. In these analyses only the information from the plain sequence was used. As a consequence there were high numbers of both false positives created by sequencing errors, and false negatives resulting from data that did not meet the conditions set by the researchers, such as minimum number of sequences or sequence similarity surrounding a SNP. Furthermore, low rates of validation were often obtained, in part because of lack of information on sequence quality and on intron position. For example, if a predicted marker spans an intron it will be difficult to detect by PCR.

There is now an increasing wealth of resources in the tomato databases and similar repositories such as sequence qualities, unigene assemblies, open reading frame (ORF) predictions, sequence similarity with other plant species and localization to genetic maps [17]. The use of this metadata allows for more sophisticated experimental designs that increase the quality of SNP prediction and information on the SNP's effects. For example, by comparing tomato and *Arabidopsis thaliana *databases a set of markers was developed that targeted loci conserved throughout evolution both in sequence and copy number [18,19]. This Conserved Orthologous Set (COS) has been proven an invaluable resource for comparative and evolutionary studies among plants [20-22]. Similarly, a recent publication used intron positions conserved between tomato and *Arabidopsis thaliana *to detect intraspecific SNPs in noncoding regions, demonstrating the possibilities of bioinformatics predictions [23].

Despite the availability of sequence-associated metadata and the need for functional genomic studies in tomato, there is surprisingly little information about the relevance and levels of variation found in coding regions. Previous SNP mining efforts are either based on noncoding regions or have no information about the effect of the polymorphisms on protein sequence. Moreover, most SNPs mined from EST sequences have little probability of being functionally important, since the majority are expected to fall in the un-translated regions (UTR), where the polymorphism rate increases more than five fold in comparison with coding regions, [23]. Information about the number, localization and type of non-synonymous polymorphisms will help unravel useful information about the evolution of genes that may have adaptive significance [24], serving as a primer for more profound studies on natural variation of interesting traits.

To perform reliable evolutionary and phylogenetic analyses sufficient polymorphism data can be obtained from the sequences of the wild species already available. However, most studies have focused on finding intraspecific molecular markers between cultivated tomato cultivars. The few attempts to infer rates of polymorphism and selection on tomato genes among its wild relatives are reduced to small sets of genes likely providing biased estimations [25].

To fill the gap in knowledge about sequence similarities and differences among cultivated and wild tomato species, we mined the EST and unigene tomato database from SGN in search for substitutions and insertions/deletions between and within the three species with highest representation: *S. lycopersicum*, *S. habrochaites *and *S. pennellii*. We used additional metadata from the database to effectively predict SNPs and infer levels of polymorphism in coding versus noncoding regions and in gene families. We surveyed this dataset for signatures of sequence evolution, selection and/or adaptation using the McDonald-Kreitman test [26] and codon-based maximum likelihood analyses [27,28]. Based on the polymorphisms detected we also developed a set of specific molecular markers and a website to make these available to the community at http://www.plb.ucdavis.edu/labs/maloof/TomatoSNP/index.html.

## Results and discussion

### EST assembly

**Figure 1:**
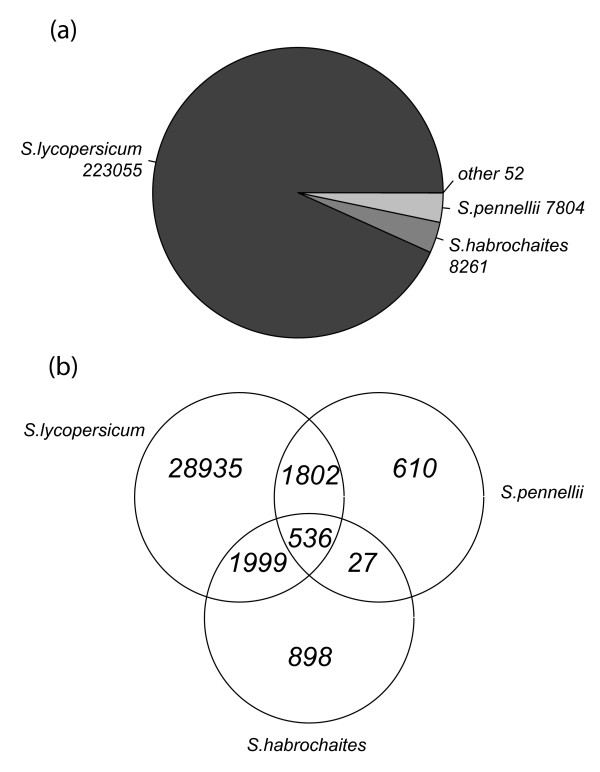
**Number, origin and distribution of ESTs in the SGN unigene collection**. (a) Number of EST included in the unigene collection at SGN divided by species of origin. Species names are followed by the number of ESTs found for each species. Other species include *S. lycopersicoides*, *S. cheesmaniae*, *S. peruvianum *and *S. pimpinellifollium*. (b) Number of unigenes with ESTs from the species analyzed in this work.

The unigene database version 200607 build 1 from SGN consists of 239,172 ESTs grouped into 34,829 unigenes, each one containing between 1 and 1087 ESTs, with an average of 6.9 ESTs per unigene (data not shown). Unigenes can be formed by EST sequences from any tomato race or species, although most unigenes (87%) contain sequences from a single species (Figure 1).

We mined sequence assemblies of *S. lycopersicum *(L) ESTs for intraspecific polymorphisms and assemblies containing sequences from both *S. lycopersicum *and *S. habrochaites *(LxH) or *S. lycopersicum *and *S. pennellii *(LxP) for interspecific SNPs. To reduce the complexity of each analysis we reassembled the unigenes using only ESTs from the species relevant to the analysis. The consensus sequence for each reassembled contig was then aligned to the original unigene sequence and, when available (88.89% of the unigenes, data not shown), to the predicted coding sequence (CDS). In the cases where a predicted CDS was available, each nucleotide in the assembly was annotated as 5' UTR, CDS or 3' UTR. We assigned quality scores to each position in the assemblies by calculating separately for each species the sum of the qualities of all nucleotides at that particular position.

**Figure 2:**
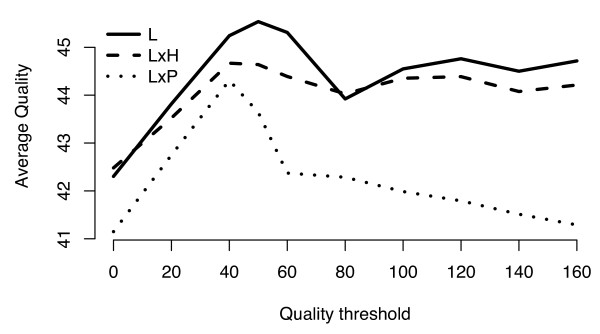
**Quality threshold estimation**. EST assemblies were analyzed at different quality thresholds by removing those positions of the assemblies whose sum of qualities were below the threshold (see Methods). The average quality of the sequences in the remaining positions was plotted (y-axis) against the range of quality thresholds tested (x-axis). L – *S. lycopersicum *assemblies, LxH – assemblies including *S. habrochaites *and *S. lycopersicum *ESTs, LxP – assemblies including *S. lycopersicum *and *S. pennellii *ESTs. Maximum average sequence quality is achieved with thresholds of 40 for LxH and LxP and 50 for the L assemblies.

Several filters were applied to diminish the number of false positive SNPs predicted in our analyses. Assembly regions that did not align with unigene sequences were removed. We also discarded positions where only a single sequence was found and, in the interspecific analyses, assembly regions where sequences from only one of the species assayed were detected. Using the remaining portions of the assemblies we determined an optimum quality threshold for each analysis at which the average sequence quality was maximized (see Methods, Figure 2). Assembly positions that did not pass the quality threshold were removed, leaving for SNP mining a total of 4,712 unigenes spanning 1,736 Kb of interspecific assemblies and 19,159 unigenes including 11,058 Kb of intraspecific *S. lycopersicum *data (Table 1).

**Table 1:** Unigenes, SNPs, and SNP rates

Analysis	LxP			LxH			L		
	
	CDS	UTR	NA	CDS	UTR	NA	CDS	UTR	NA
Unigenes analyzed		2249			2463			19159	
Kb analyzed	504.9	116.8	21.5	860.1	203.6	29.7	8446.3	1962.1	649.6
	3728/	1104/		8457/	3501/	349/	14455/	8229/	919/
SNPs/indels	206	534	92/30	614	1611	64	1980	3006	221
Unigenes with SNPs		1341			1934			5084	
SNP rate		0.8852			1.3349			0.2605	
SNP rate per region	0.779	1.4025	0.5682	1.0546	2.5107	1.3925	0.1946	0.5726	0.1755

Only nonconsecutive SNPs are displayed. NA- gene region is not available for unigenes in which a CDS could not be predicted. SNP rates are calculated as the number of SNPs per 100 bp.

**Figure 3:**
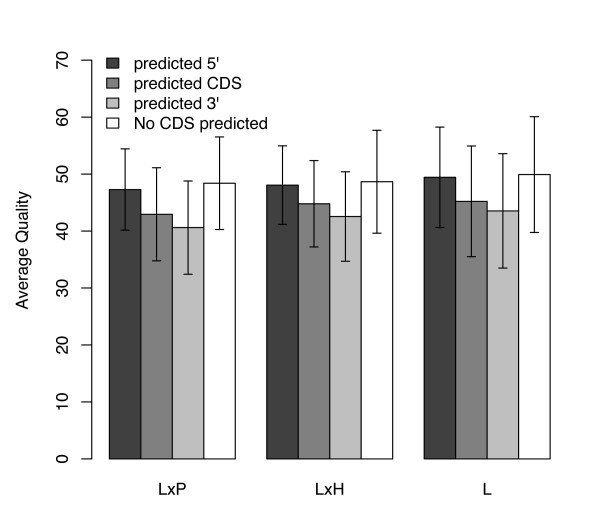
**Differences in average sequence quality between gene regions**. Average quality of the sequences mined for SNPs in each analysis grouped by estimated gene region. Error bars represent standard deviation. LxP – Assemblies including *S. lycopersicum *and *S. pennellii *ESTs, LxH – Assemblies including *S. habrochaites *and *S. lycopersicum *ESTs, L – *S. lycopersicum *assemblies.

In every analysis the majority of nucleotides assembled were located in predicted coding regions. Between 2.8 and 6.2% of the positions could not be assigned to a gene region due to the lack of predicted CDS (Table 1). The average quality of the nucleotides considered in the assemblies was always highest in the 5' regions and lowest in the 3' regions (Figure 3). This could be explained by the bias towards sequencing the ESTs from the 5', as an average of 95.87% of the sequences from each library were readings from the top strand (data not shown). Interestingly, assemblies from unigenes for which there is no predicted CDS had overall average qualities similar to the 5' region (Figure 3). This raises the possibility that those unigenes contain mostly 5' UTR sequence, in turn explaining why ESTscan [29,30] was not able to predict a CDS.

### SNP mining

**Figure 4:**
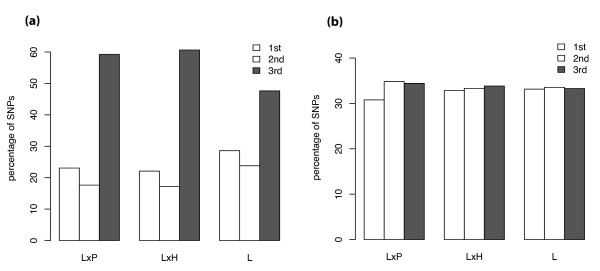
**Percentage of polymorphism in each codon position**. Percentage of total SNPs found at each codon position in the assayed regions. (a) Assembly positions falling in predicted coding regions were assigned to the first, second or third codon positions. The percentage of non-consecutive SNPs found in each codon position is shown. (b) Codon positions in the UTRs were calculated by extrapolating the predicted ORF, and the percentage of non-consecutive SNPs found in each hypothetical codon position is shown.

We mined the resulting sequence assemblies for nonconsecutive SNPs taking into account the position of the SNP with respect to the predicted gene regions. In total 40,834 substitutions and 8,266 insertions/deletions (indels) were detected (summarized in Table 1; see Additional files 1, 2, 3 &4 for full data). Several observations confirm the performance of our SNP detection algorithms. As shown in Table 1, indels appear between 2.6 and 8.7 times more frequently in UTRs than in the CDS. Insertion and deletion of nucleotides are likely to produce changes in the open reading frames disrupting correct translation, therefore selection against them is expected in coding regions. Similarly, predicted noncoding regions yielded between 1.8 and 2.9 times more SNPs per kilobase analyzed than coding regions (Table 1), as reported in previous tomato analyses [15,19,23,25]. We next used the estimated CDS for each unigene to calculate the codon position for each SNP. In translated regions the majority of the mutations are located in the third position of the codon (Figure 4). This result was expected since selective pressure in coding regions reduces the number of non-synonymous substitutions and the redundancy of the genetic code is mainly in the third base of the codons. We then calculated hypothetical codons in the UTRs by extending the ORF from the origin of transcription in the predicted CDS. The third position bias disappeared in these hypothetical noncoding regions (Figure 4) suggesting that on average the SNP, CDS, and codon position predictions are accurate. In addition, when looking at all SNPs in coding regions together, the percentage of non-synonymous SNPs is 46.37%, similar to genome-wide analyses in Arabidopsis (45.34%) [31] and humans (46.46%) [32].

Regarding the differences between intra and interspecific analyses, we found only 26.5% of the analyzed loci to be polymorphic when mining a single species versus the 69.5% when analyzing pairs (Table 1). For interspecific analysis the SNP rates were comparable to those published before between *S. pennellii *and *S. lycopersicum *(1.02 to 1.61 SNPs/100 bp) making no distinction between coding and non coding regions [14,33]. The same holds true for *S. lycopersicum *intraspecific analyses, where reported SNP rates range between 0.0117 and 0.585 SNPs/100 bp on all EST sequences analyzed regardless of their location in the gene [14-16,25].

### SNP representation in Gene Ontology classes

**Figure 5:**
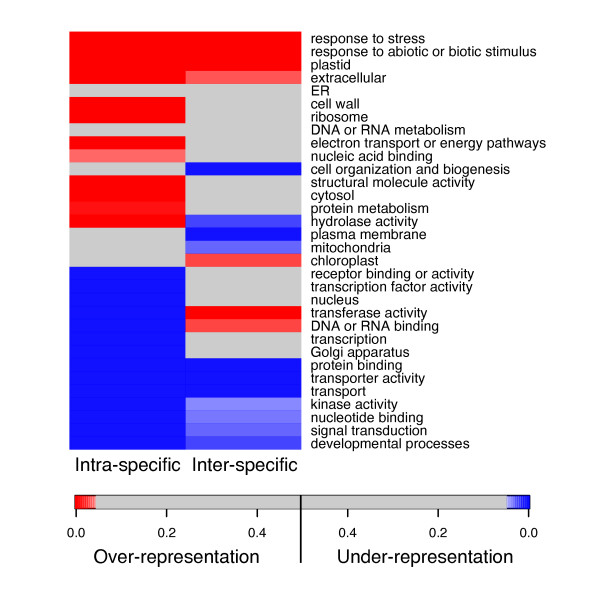
**Polymorphism occurrence by Gene Ontology categories**. GO over- and under-representation was calculated using the number of non-synonymous SNPs and the total number of nucleotides analyzed in predicted coding regions. Interspecific analysis was performed pooling the unigenes from the LxH and the LxP analyses as described in Methods. Red boxes indicate over-representation of SNPs in a specific GO category at p < 0.05 and blue boxes under-representation at p < 0.05.

We reasoned that certain gene families might be more variable among tomato cultivars and species than others. For example, since tomato species are adapted to diverse environments, genes involved in environmental response might accumulate a higher number of non-synonymous SNPs due to selection. We used Gene Ontology (GO) categories [34], which group genes into functionally related classes, to assess the differential polymorphic rates of unigenes encoding specific classes of proteins. We assigned GO categories to each nucleotide position in the assemblies based on the closest *Arabidopsis thaliana *homolog, and looked for categories with over- or under-representation of non-synonymous SNPs. To achieve higher power we grouped together the data from the interspecific analyses. The larger number of sequences available in the intraspecific analysis allowed greater statistical power, although the most over- and underrepresented gene classes are in agreement in both analyses (Figure 5). As expected, GO categories related to environment interaction, such as responses to stress and abiotic stimulus, had an over-representation of non-synonymous polymorphisms both between and within species (p < 0.001, Figure 5). On the other hand, categories involved in basic biological processes and transcription regulation showed a relative lack of polymorphisms, as had been found in *Arabidopsis thaliana *accessions [31]. Surprisingly, genes encoding ribosomal proteins also presented more SNPs that expected, perhaps due to the challenge of distinguishing homologs from paralogs in this gene family, which shows complex patterns of copy number variants in Arabidopsis [35].

### McDonald-Kreitman test

The neutral evolution theory holds that most within species polymorphisms and between species differences have little fitness consequence [36]. As a result, the ratio of non-synonymous to synonymous substitutions should be similar for a particular gene both within and between species. Deviations from this can be a sign of non-neutral evolution [26]. To examine this prediction, we constructed alignments of the wild and cultivated consensus sequences containing only the good quality positions and the SNPs predicted by our algorithms, and surveyed each alignment for signatures of positive selection using the McDonald-Kreitman test [26]. With this method we tested 1425 unigenes from the *S. lycopersicum *and *S. habrochaites *analysis and 924 unigenes from the *S. lycopersicum *and *S. pennellii *analysis. Before correction for multiple testing we found significant excess of non-synonymous mutations (p < 0.05) in 16 unigenes in the LxH analysis and 3 unigenes in the LxP analysis (data not shown). However, none of those unigenes survived the Benjamini and Hochberg correction for multiple testing [37].

### Maximum likelihood codon-substitution models

The McDonald-Kreitman test compares variation between and within two species. An alternative approach is to simultaneously analyze all the sequences in a phylogenetic tree [38]. Following this method, we surveyed the unigenes for signals of positive selection using maximum likelihood estimates from codon-substitution models [27,28]. We reasoned that genes important for domestication might show a higher rate of non-synonymous to synonymous substitutions specifically in the *S. lycopersicum *lineage. To test this idea, we fit two different models for each unigene using alignments of the coding regions from cultivated tomato, *Arabidopsis thaliana *and *S. habrochaites *or *S. pennellii*. One of the models used assumed similar non-synonymous/synonymous rates (d_n_/d_s_) in every branch of the evolutionary tree (one-branch rate model). The second model allowed for different d_n_/d_s _estimates in the branch leading to *S. lycopersicum *relative to the rest of the tree (two-branch rates model). For each gene we used a likelihood ratio test to ask if the two-branch model fit significantly better than the one-branch model. Support for two-branch rates is suggestive of directional selection on the branch leading to domesticated tomato, therefore raising the possibility of identifying genes important for domestication [38]. Alternatively, *S. lycopersicum*-specific d_n_/d_s _ratios could occur due to natural selection acting after the *S. lycopersicum *lineage split from *S. pennellii *or *S. habrochaites *but before domestication. We were able to test 1682 unigenes from the *S. lycopersicum *and *S. habrochaites *analysis and 1384 unigenes from the *S. lycopersicum *and *S. pennellii *analysis. From those, 9 and 1 unigenes respectively presented evidence of elevated non-synonymous substitution rates on the branch leading to *S. lycopersicum *after correction for multiple testing (Table 2), suggesting these genes as possible targets of selection during domestication. We cannot discard, however, the possibility that some or all of these genes have an excess of fixed polymorphisms in the cultivated tomato lineage due to a population bottleneck during domestication [39], or because of the effect of natural selection due to differing environments. Among these genes we found that the three GO categories most represented were responses to abiotic and biotic stimulus, responses to stress and protein metabolism. This finding is in concordance with our analysis of SNP over-representation in gene families.

**Table 2:** Unigenes under positive selection detected by the likelihood codon substitution models.

Analysis	Unigene	Annotation^1^	p-value^2^	Sites^3^	d_n_/d_s_^4^	d_n_/d_s _1^5^	d_n_/d_s _2^6^	lnL^7^	lnL2^8^
LxH	SGN-U314303	Aldehyde dehygrogenase	0.00132	491	0.0665	0.0343	998.99	-3184.6	-3173.1
LxH	SGN-U317105	Unknown protein	0.00132	286	0.2677	0.0058	145.99	-1821.6	-1810.1
LxH	SGN-U315058	Tropinone reductase	0.00211	275	0.1361	0.0836	998.99	-1902.2	-1891.5
LxH	SGN-U315796	Alcohol dehydrogenase	0.00276	399	0.0924	0.0036	0.35	-2899.9	-2890.1
LxH	SGN-U317449	Fe(II)/ascorbate oxidase	0.00276	441	0.0569	0.0019	193.75	-2443.7	-2433.8
LxH	SGN-U317968	Hypothetical protein	0.00276	253	0.2771	0.0053	3.34	-1058.9	-1049.1
LxH	SGN-U319889	Putative kinesin light chain	0.00941	437	0.1566	0.0032	121.86	-1966.2	-1957.8
LxH	SGN-U314953	Unknown protein	0.01001	176	0.2633	0.0485	998.99	-1305.9	-1297.6
LxH	SGN-U318509	Hypothetical protein	0.03006	547	0.1026	0.0172	0.52	-2158.9	-2151.8
LxP	SGN-U314632	Ubiquitin extension protein 2/60S ribosomal protein L40	0.00099	155	0.0580	0.0017	998.99	-512.5	-500.2

LxP – *S. lycopersicum *and *S. pennellii *analysis, LxH – *S. lycopersicum *and *S. habrochaites *analysis. ^1^Annotation – based on the annotation from the best BLAST hit in *Arabidopsis thaliana*. ^2 ^p-value – Multiple testing corrected p-values for the differences in the maximum likelihood estimates of the two models fit. ^3 ^Sites – number of codons analyzed, ^4 ^d_n_/d_s _– d_n_/d_s _for the model where one d_n_/d_s _ratio is estimated for the entire evolutionary tree. ^5 ^d_n_/d_s _1 – d_n_/d_s _for the non *S. lycopersicum *lineages in the model where a different d_n_/d_s _ratio is estimated for the branch leading to *S. lycopersicum*. ^6 ^d_n_/d_s _2 – d_n_/d_s _for the *S. lycopersicum *lineage in the model with two d_n_/d_s _ratios. The very high d_n_/d_s _ratios observed for some genes are a consequence of relatively little diversity between wild and domesticated tomato and are likely to be an overestimate of the true rate. ^7 ^lnL – likelihood estimate for the model with one d_n_/d_s _ratio. ^8 ^lnL2 – likelihood estimate for the model with two d_n_/d_s _ratios.

### Molecular marker design

Using the information gathered from the assemblies we developed a set of molecular markers for detecting the predicted SNPs. Successful design of PCR based molecular markers from EST sequences requires the ability to avoid amplifying introns. Since many Arabidopsis intron positions are conserved in tomato [23], we used this information (see Methods) to inform the design of molecular markers for the polymorphisms identified in the interspecific analyses. First, we tested our intron predictions by designing three primer pairs surrounding predicted introns. One of those produced a band 700 bp greater than expected without introns and the other two did not amplify, suggesting the existence of introns where predicted (data not shown). We did not carry out similar analysis for the intraspecific SNPs due to the lack of information within SGN regarding the *S. lycopersicum *accessions used. Although it is well known that *S. lycopersicum *ESTs in SGN come from several cultivars, the information for each individual EST library is not available. For each high-quality, interspecific SNP we developed a database containing suggested primers to amplify the SNP region, restriction enzymes to detect the polymorphism, and predicted fragment sizes before and after digestion of each allele. This information can be accessed at http://www.plb.ucdavis.edu/labs/maloof/TomatoSNP/index.html.

We validated a subset of markers by using 491 primer pairs designed to amplify fragments from *S. lycopersicum *and from *S. pennellii*, *S. habrochaites*, or both. We calculated the size differences between the amplified products cut with the appropriate restriction enzymes when needed or uncut in the case of indels. From the 491 primer pairs, 6 pairs amplified fragments that were smaller than expected and were discarded. Another 48 pairs failed to amplify fragments in at least one species, leaving us with 437 primer pairs that would test 281 polymorphisms between *S. lycopersicum *and *S. habrochaites *and 228 polymorphisms between *S. lycopersicum *and *S. pennellii*. Among these, 30 primers pairs yielded bands that were bigger than expected, probably due to the existence of non-conserved introns, nevertheless 23 of these were polymorphic as expected after restriction enzyme digestion. For the remaining amplifications we found 87% (220 of 261 LxH and 193 of 210 LxP) of the molecular markers to work as predicted. It is worth noting that 10% of the successful markers showed bands corresponding to both alleles in at least one of the species tested, suggesting heterozygosity in the lines used or the amplification of fragments from a family of genes sharing that sequence. Markers that failed could be due to undetected SNPs in the sequence that modify the predicted restriction sites, or errors in SNP prediction. All tested markers are available in the Additional file 5.

## Conclusion

We report in this work more than forty-nine thousand inter and intraspecific polymorphisms mined from the EST databases of the cultivated and two wild species of tomato. By taking advantage of the additional information linked to each sequence we were able to more accurately estimate the quality and the position of each SNP with respect to the coding region, which allowed us to distinguish those polymorphisms more likely to have phenotypic effects. Comparison of the sequences to homologs from better characterized species also allowed us to functionally classify the predicted unigenes and perform gene evolution analysis and tests for positive selection. We were able to suggest candidate genes and gene families that may be related to domestication of the cultivated tomato and/or environmental adaptation of wild species, providing hypotheses for more involved evolutionary studies. The information obtained was also used to design a set of markers that we make available to the community via a website. To our knowledge this is the first time that substantial meta-information including quality values, open reading frame predictions and homology to genes in *Arabidopsis thaliana*, has been used on tomato sequences to perform a pre-genomic analysis of gene variability and evolution.

## Methods

### Sequence data

Fasta files containing version 200607 build 1 of the unigene sequences, EST sequences, EST qualities and estimated coding region for each unigene were downloaded from SGN. ESTs in this database originated from the sequencing of at least 43 *S. lycopersicum *cDNA libraries belonging to at least two different accessions, 2 *S. pennellii *and 2 *S. habrochaites *cDNA libraries plus some individual cDNAs. Unigenes are the consensus sequences of these ESTs assembled with cap3 software as described in [17]. CDSs for the unigenes were calculated by SGN with ESTscan software [29,30]. This software returns an optimum open reading frame based on Markov models and the nucleotide usage found in coding regions. Lukas Mueller at SGN kindly generated a custom list of all the unigenes and their constituent ESTs. The highest BLAST hit of every unigene versus the *Arabidopsis thaliana *genome was bulk queried and downloaded from SGN. Arabidopsis thaliana sequences were downloaded from TAIR (version 20080412, http://www.arabidopsis.org)

### Assembly construction

We mined intraspecific SNPs in *S. lycopersicum *(L) ESTs and interspecific SNPs between *S. lycopersicum *and *S. habrochaites *(LxH) and between *S. lycopersicum *and *S. pennellii *(LxP) ESTs. Intraspecific analysis of *S. pennellii *and *S. habrochaites *ESTs were not performed as the EST libraries for those species were developed from a single accession.

For each unigene we used cap3 with relaxed parameters (-p 66 -b 99 -e 200) to assemble the EST sequences from the species participating in each of the analyses into analysis-specific contigs. Using these assemblies we calculated the sum and average of the qualities for each nucleotide call at every position and obtained analysis-specific consensus sequences. These consensus sequences were aligned to the original unigene sequence and the predicted CDS to estimate the beginning and end of the coding region. Every step in this process was carried out and verified using custom Perl scripts.

Perl and R [40] scripts were developed to estimate the quality score thresholds for sequence inclusion in SNP discovery. Once a threshold was determined we exclusively considered the positions in the assemblies above the threshold as follows. For interspecific analyses we required the sum of all the qualities of the nucleotides from each species to be over the threshold. For the intraspecific analysis we developed an algorithm that partitions all qualities at a given position into two groups with the minimum difference of the sums. We then considered only those positions in the assemblies at which the sum of the qualities of both groups was over the threshold. The threshold was defined as the quality score that maximizes the average sequence quality calculated separately for each species (interspecific analyses), or for each group of qualities (intraspecific analysis) taking into account only those positions over the threshold (Figure 2). We determined a quality threshold score of 50 for the intraspecific *S. lycopersicum *analysis and of 40 for both interspecific analyses (Figure 2).

### SNP discovery

We defined SNPs in the intraspecific analysis as any assembly position where two and only two different bases were registered and for which the sum of qualities for each nucleotide call was over the quality threshold imposed for the analysis. For the interspecific analysis we considered SNPs whose positions within the assemblies presented a single and different nucleotide call for each species, and whose sum of qualities was greater than the imposed quality threshold. For quantification, SNPs that were consecutive in the assemblies were counted as a single polymorphisms. SNP rates were calculated as the number of non-contiguous SNPs per 100 bases.

Amino acid translation, SNP codon position and transitions/transversion ratios were evaluated with custom Perl and R scripts that analyzed the EST assemblies, the unigene sequence and the predicted CDS sequences.

### Differential representation of SNPs in GO terms

Each nucleotide in the EST assemblies was assigned one or more GO categories based on the terms from the homologous *Arabidopsis thaliana *locus. To increase the accuracy of the test, we used only the parts of the assemblies that corresponded to coding regions and only those SNPs that has been predicted to produce amino acid changes. GO categories text files (version 20080712) were downloaded from TAIR [34]. For the interspecific analyses, we pooled the nucleotide calls for both (LxH and LxP) analyses. For duplicate loci we removed the locus with the shortest sequence. R scripts were developed to calculate over and under-representation of SNPs in nucleotide pools of each GO category versus all SNPs/nucleotides detected in the analysis using Fisher's exact test.

### Tests for selection

We performed McDonald-Kreitman tests and estimated Maximum likelihood from codon-substitution models on those unigenes that contained *S. lycopersicum *together with *S. pennellii *or *S. habrochaites *sequences. For the McDonald-Kreitman test we built fasta files for each unigene with the estimated CDS for the wild species alleles and two *S. lycopersicum *alleles differing in the SNPs identified in the L analysis. To maintain the fidelity of the analysis, those positions that did not pass the quality threshold using the methods described above were substituted with the 'unknown' character and not considered in the subsequent analysis. The number of synonymous and non-synonymous substitutions and p-values were calculated using the previously described MK.pl Perl script [41].

Two maximum likelihood codon-substitution models were fit to test the hypothesis of existence of positive selection in each unigene [27,28]. First, we fit the null model with a single d_n_/d_s _ratio with equal ratios in every branch. The second model allowed for two d_n_/d_s _ratios: one for the *S. lycopersicum *lineage and one for the rest of the tree. Then, a likelihood ratio test of the hypothesis of two branch rates was calculated by comparing the likelihood values from both models as in [42]. To do this we created Perl scripts that used ClustalW [43] to align the cultivated and wild species predicted CDS and the homologous *Arabidopsis thaliana *coding sequences. Alignments whose sum of qualities were not over the imposed quality threshold were substituted with the 'unknown' character. We also removed the parts of the alignments that lacked sequences from any of the three species. We constructed a phylogenetic tree of the three species and used PAML (v 3.14 [44]) to calculate the maximum likelihood of the models.

The resulting p-values from these experiments were corrected for multiple testing using the Benjamini and Hochberg algorithm in the Bioconductor multtest package [37,45].

### Molecular marker design

For each polymorphic unigene in the interspecific analysis we aligned its predicted protein sequence with the protein sequence of its Arabidopsis best BLAST hit using standalone BLAST and custom Perl scripts. We calculated intron positions in the unigene based on intron positions in the Arabidopsis CDS. For each SNP we designed primers using Primer3 [46] with the unigene sequences as input, adjusting the program to design the primers within the predicted exon where the SNP was located.

We used Bioperl to generate virtual PCR fragments for each SNP allele based on the primers designed, and to find restriction endonucleases that would differentially cut the fragments, thus creating molecular markers. A set of these molecular markers was tested on genomic DNA from *S. lycopersicum *VF36, *S. pennellii *LA716 and *S. habrochaites *LA1347. Touchdown PCR was performed in a MJ Research PTC-200 Thermocycler with a starting annealing temperature of 58°C, which decreased 0.5°C per cycle for 15 cycles and stayed constant at 55°C for 30 cycles. Extension time was 40 seconds and denaturizing steps were performed for 30 seconds at 96°C. PCR products were digested with the appropriate restriction enzymes to detect the polymorphisms. We developed a database and a website holding the molecular marker information for each interspecific SNP.

All R and Perl scripts are available by request.

## Abbreviations

CDS: Coding sequence; COS: Conserved orthologous set; EST: Expressed sequence tag; GO: Gene ontology; L: *S. lycopersicum *intraspecific analysis; LxH: *S. lycopersicum *versus *S. habrochaites *interspecific analysis; LxP: *S. lycopersicum *versus *S. pennellii *interspecific analysis; ORF: Open reading frame; PCR: Polymerase chain reaction; RFLP: Restriction fragment length polymorphism; SGN: Solanaceae genomics network; SNP: Single nucleotide polymorphism; UTR: Untranslated regions.

## Authors' contributions

JMJG wrote the manuscript, conceived and designed the study and the website hosting the results. JMJG also performed the assemblies, SNP mining, evolutionary analysis and molecular marker design and essays. JNM conceived and participated in the design of the study, and helped to draft the manuscript. All authors read and approved the final manuscript.

## Supplementary Material

Additional file 1 **Non-synonymous SNPs**. Consecutive and non-consecutive non-synonymous polymorphisms. "Analysis" Column, LxP: *S. lycopersicum *and *S. pennellii *interspecific analysis, LxH: *S. lycopersicum *and *S. habrochaites *interspecific analysis. L: *S. lycopersicum *intraspecific analysis. "Position" indicates the nucleotide position in the unigene sequence from the SGN database. "Nt1" and "Nt2": Nucleotide call (reverse nucleotide if the sequence is in reverse orientation). "# of Seqs 1" and "# of Seqs 2": number of ESTs in the assembly with that nucleotide call. "Quality Sum 1" and "Quality Sum 2": sum of qualities of all nucleotides with that call. "CDS Orientation": U if plus strand, C if minus strand. "Codon 1" and "Codon 2": estimated codon including the SNP, "Amino Acid 1" and "Amino Acid 2": translation for Codon 1 and Codon 2 respectively. For all headings "1" refers to cultivated tomato in every analysis whereas "2" indicates the wild species in the interspecific analyses and a second allele of cultivated tomato in the intraspecific analysis. Click here for file

Additional file 2 **Synonymous SNPs**. Consecutive and non-consecutive synonymous polymorphisms. Legend as in Additional file 1 Click here for file

Additional file 3 **SNPs affecting stop codons**. Consecutive and non-consecutive polymorphisms causing premature stop codons or disrupting stop codons. Legend as in Additional file 1 Click here for file

Additional file 4 **SNPs in UTRs**. Consecutive and non-consecutive polymorphisms located in the predicted UTR regions. "Region" indicates if the SNP was found in the 3' or 5' UTR region. Other headings as in Additional file 1. Click here for file

Additional file 5 **Molecular markers experimentally tested**. Molecular markers that returned amplification products both polymorphic and non-polymorphic. "MNS", minimum number of sequences of any species at the SNP position; "MQS", minimum average sequence quality at the SNP position (See Methods). In the column names, L stands for *S. lycopersicum *and W for the wild species *S. pennellii *or *S. habrochaites*. "PREDICTED_RESTRICTION_SIZES" gives the sizes of the predicted fragments after digestion, separated by underscores if multiple bands are predicted. "COMMENTS": 'ok' if the sizes were as expected, 'extra bands' if additional bands amplified in addition to the ones expected, 'good pattern' if the sizes of the amplifications obtained were different than expected but the polymorphism was recognizable after restriction, 'het/gene family' if at least one of the parents presented both alleles at the same time, or empty is the polymorphism was not found. Click here for file
